# Supplementary material for: Superconducting/magnetic Three-state Nanodevice for Memory and Reading Applications
Source: Sci Rep. 2015 Oct 15;5:15210. doi: 10.1038/srep15210 (PMC4606737; doi:10.1038/srep15210)
Supplement: Supplementary Information [file srep15210-s1.pdf]

SUPPLEMENTARY INFORMATION

Superconducting/magnetic three-state nanodevice for memory and reading applications

J. del Valle[1], A. Gomez[1,+], E. M. Gonzalez[1,2], M. R. Osorio[2], D. Granados[2] and J. L. Vicent[1,2*]

[1]Departamento Fisica de Materiales, Facultad de CC. Fisicas, Universidad Complutense, 28040 Madrid (Spain).

[2]IMDEA-Nanociencia, Cantoblanco, 28049 Madrid (Spain)

[+] Present address: Centro de Astrobiología (CSIC-INTA), Torrejón de Ardoz, 28850 Madrid (Spain).

I. MAGNETIC CHARACTERIZATION OF THE Co/Pd NANOTRIANGLE

Magnetic multilayers based on Co/Pd system allows fabricating nanomagnets with different remanent magnetic states, including magnetic vortex state, in plane magnetic state, and out of plane magnetic states, only by changing the layer thicknesses (see Ref. S1).

In this work, we have grown Co (0.4 nm) / Pd (0.6 nm) multilayers using magnetron sputtering on Si substrate (see Methods).

Figure S1 shows Co/Pd multilayer hysteresis loops of both the unpatterned film and the nanotriangle array, obtained using SQUID magnetometer. As can be seen, the unpatterned film retains full perpendicular magnetization at H=0. It can be noted that the triangles' remanent magnetization is also high, around $0.85M_S$, and coercive field is increased with respect to the Co/Pd film.

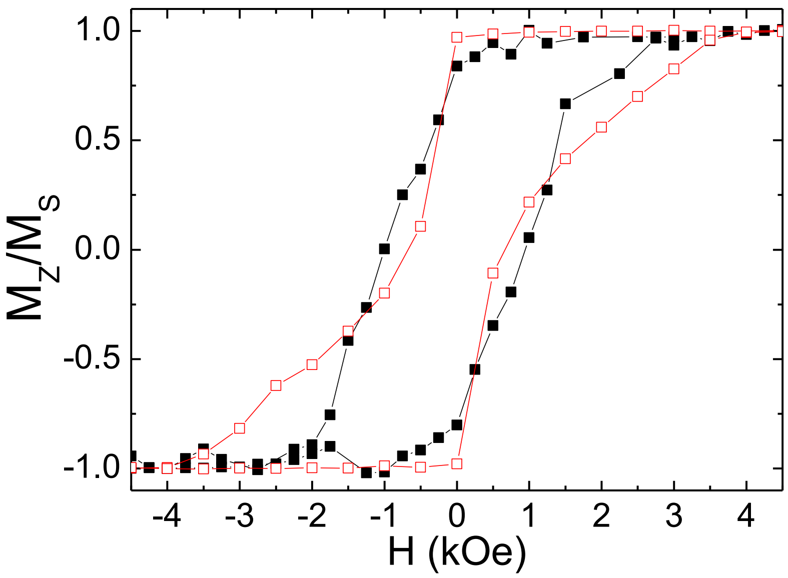

Figure S 1. Out of plane normalized hysteresis loops for the Co/Pd film (hollow red squares) and the array of Co/Pd triangles (black squares).

II. MAGNETORESISTANCE MINIMA AND MATCHING EFFECTS

The matching magnetic fields B_m (resistivity minima) can be calculated taking into account that they are evenly separated and that the main matching field (*m*=1) is the one for which the density of vortex lattice equals the density of pinning centers (see Ref. S2), where *n* is the density of pinning centers:

$$B_m = m \cdot n \cdot \Phi_0 \qquad (1)$$

The values of the matching fields can be calculated straightforwardly. We present below examples taken from the main regular arrays:

Triangular lattice array (with *a* being the side of the equilateral triangle):

$$n(tri-lattice) = \frac{1/2}{(\sqrt{3}/4)\cdot a^2} = \frac{2}{\sqrt{3}\cdot a^2} \cong \frac{1.155}{a^2}$$

$$B_m(tri-lattice) = m \cdot \frac{1.155}{a^2} \cdot \Phi_0 \qquad (2)$$

Square lattice array (with *a* being the side of the square):

$$n(sq-lattice) = \frac{1}{a^2}$$

$$B_m(sq-lattice) = m \cdot \frac{1}{a^2} \cdot \Phi_0 \qquad (3)$$

Rectangular lattice array (with *a, b* being the sides of the rectangle):

$$n(rect-lattice) = \frac{1}{ab}$$

$$B_m(rect-lattice) = m \cdot \frac{1}{ab} \cdot \Phi_0 \qquad (4)$$

Supplementary reference

S 1 Gomez A., Gilbert D. A., Gonzalez E. M., Liu K., Vicent J. L. Control of dissipation in superconducting films by magnetic stray fields. *Appl. Phys. Lett*. **102**, 052601 (2013).

S 2 Gomez A., Gonzalez E. M., Vicent J. L. Superconducting vortex dynamics on arrays with bicrystal-like structures: matching and rectifier effects. *Supercond. Sci. Technol.* **25**, 124006 (2012).
